# Comprehensive profiling of DNA methylation in colorectal cancer reveals subgroups with distinct clinicopathological and molecular features

**DOI:** 10.1186/1471-2407-10-227

**Published:** 2010-05-21

**Authors:** Pei Woon Ang, Marie Loh, Natalia Liem, Pei Li Lim, Fabienne Grieu, Aparna Vaithilingam, Cameron Platell, Wei Peng Yong, Barry Iacopetta, Richie Soong

**Affiliations:** 1 School of Surgery, University of Western Australia, Perth, Australia; 2 Cancer Science Institute of Singapore, National University of Singapore, Singapore, Singapore; 3 Department of Haematology and Oncology, National University Hospital, Singapore, Singapore; 4 Department of Surgery, St John of God Hospital, Subiaco, Australia

## Abstract

**Background:**

Most previous studies of the CpG island methylator phenotype (CIMP) in colorectal cancer (CRC) have been conducted on a relatively small numbers of CpG sites. In the present study we performed comprehensive DNA methylation profiling of CRC with the aim of characterizing CIMP subgroups.

**Methods:**

DNA methylation at 1,505 CpG sites in 807 cancer-related genes was evaluated using the Illumina GoldenGate^® ^methylation array in 28 normal colonic mucosa and 91 consecutive CRC samples. Methylation data was analyzed using unsupervised hierarchical clustering. CIMP subgroups were compared for various clinicopathological and molecular features including patient age, tumor site, microsatellite instability (MSI), methylation at a consensus panel of CpG islands and mutations in *BRAF *and *KRAS*.

**Results:**

A total of 202 CpG sites were differentially methylated between tumor and normal tissue. Unsupervised hierarchical clustering of methylation data from these sites revealed the existence of three CRC subgroups referred to as CIMP-low (CIMP-L, 21% of cases), CIMP-mid (CIMP-M, 14%) and CIMP-high (CIMP-H, 65%). In comparison to CIMP-L tumors, CIMP-H tumors were more often located in the proximal colon and showed more frequent mutation of *KRAS *and *BRAF *(*P *< 0.001).

**Conclusions:**

Comprehensive DNA methylation profiling identified three CRC subgroups with distinctive clinicopathological and molecular features. This study suggests that both *KRAS *and *BRAF *mutations are involved with the CIMP-H pathway of CRC rather than with distinct CIMP subgroups.

## Background

DNA hypermethylation-induced gene silencing is a common event in many malignancies and serves as an alternative mechanism to genetic mutation for the loss of tumor suppressor functions [1,2]. Although the mechanisms that underlie aberrant DNA methylation in cancer cells remain to be elucidated, current evidence suggests that it may be an early and possibly even an initiating event in the development of colorectal cancer (CRC).

A subset of CRC has been shown to exhibit frequent and concurrent hypermethylation at specific gene promoters and is referred to as the CpG island methylator phenotype (CIMP+) [3]. CIMP+ CRC is associated with distinct clinicopathological and molecular features including proximal tumor location, preponderance in elderly females, poorly differentiated and mucinous tumor histology, microsatellite instability (MSI) and frequent *BRAF *V600E mutation [3-10]. CIMP+ CRC often lack the hallmark genetic alterations in *APC*, *p53 *and 18q that characterize the classic adenoma-carcinoma sequence. Instead, CIMP+ tumors are thought to develop along an alternate serrated adenoma pathway in which hypermethylation rather than mutation is used to inactivate tumor suppressor genes [11].

In an effort to establish CIMP+ CRC as a distinct subgroup of CRC, Laird and colleagues analysed the methylation of 195 individual gene promoter regions in 295 CRC using the quantitative MethyLight assay [10]. From their results, they proposed a panel of 5 CpG island methylation markers to standardize the classification of CIMP+ CRC. However, different groups have continued to use a variety of methylation markers to define CIMP+ CRC [7,12-14]. The lack of consensus markers has led to reports of several CIMP subgroups according to the frequency of CpG island methylation [13-15]. The investigators who originally proposed CIMP recently described two subgroups of CIMP+, termed CIMP-1 and CIMP-2, that displayed increased frequencies of *BRAF *and *KRAS *mutations, respectively [14]. Similarly, Nagasaka *et al *described two distinct patterns of gene methylation in CRC that also segregated with *BRAF *and *KRAS *mutations [13,16]. Using a panel of 8 methylation markers, Ogino *et al *identified a CRC subgroup which they termed CIMP-low that was associated with frequent *KRAS *mutation, *MGMT *methylation and occurrence in males [17].

Most previous studies of CIMP+ CRC have investigated a relatively small number of CpG island markers for methylation. The GoldenGate Methylation BeadArray (Illumina, Inc.) technology provides the opportunity for high-throughput methylation analysis of a large number of CpG sites. In the present study the GoldenGate Methylation Cancer Panel I containing 1,505 CpG loci within 807 cancer-related genes was used to study methylation patterns in 91 unselected CRC. These genes were selected based on their involvement in cell growth control, differentiation, migration, apoptosis, DNA damage repair and oxidative metabolism. The GoldenGate technology allowed us to identify three distinct CRC subgroups according to their methylation pattern which showed distinctive clinicopathological and molecular characteristics and differed in their frequencies of *BRAF *and *KRAS *mutation.

## Methods

### Tissue samples

Unselected cases of CRC and adjacent normal colonic mucosa were obtained from 91 patients undergoing surgical resection at St John of God Hospital, Subiaco, Western Australia. All samples were snap-frozen in liquid nitrogen at the time of surgery and stored at -80°C until use. This set of tumors contains well-annotated clinicopathological information including age, gender, tumor location, staging, presence of lymphocytic infiltration and careful pathological assessment of perineural (PNI), lymphovascular (LVI) and extramural invasion (EMVI). Informed consent was obtained from all patients and the project was approved by the Human Research Ethics Committee of St John of God Hospital.

### BRAF mutation, KRAS mutation and microsatellite instability

DNA was extracted from approximately 25 mg of tissue using standard phenol-chloroform extraction. Hotspot mutations in *BRAF *(V600E) and *KRAS *(codons 12 and 13) were identified using fluorescent single strand conformation polymorphism (F-SSCP) as described previously [18,19]. Deletions in the BAT-26 mononucleotide repeat were detected using F-SSCP and this was used to establish MSI+ status [20].

### MethyLight determination of CIMP^W ^status

Sodium bisulfite modification was performed using the EZ DNA methylation kit according to the manufacturer's instructions (Zymo Research, Orange, CA) and eluted into 20 μl of 10 mmol/L Tris-HCl (pH 8). The required amount of genomic DNA to ensure reliable evaluation of DNA methylation following bisulfite modification was determined as described previously [21]. DNA methylation levels for the panel of markers (*RUNX3, CACNA1G, IGF2, NEUROG1, SOCS1*) described by Weisenberger *et al *[10] were measured using MethyLight as described by the authors. The percentage of methylated reference (PMR) was calculated and normalised against β-actin to account for variability in the amount of input bisulfite-treated DNA. SssI methylase-treated DNA was used as the methylated standard. A threshold PMR value of > 4 was used to classify loci as methylated or non-methylated. In the present study, CIMP^W ^refers to the classification of CIMP using the panel of markers described by Weisenberger *et al*., whereby CIMP^W^-high is defined as 3 or more methylated loci, CIMP^W^-low as 1 or 2 methylated loci and CIMP^W^-negative as no methylated loci.

### DNA methylation profiling using Illumina GoldenGate^® ^methylation bead array

Comprehensive DNA methylation profiling using the Illumina Goldengate Methylation Arrays^® ^(Illumina, San Diego, CA) was carried out as described by Bibikova *et al *[22] on 91 CRC and 28 randomly selected, matched normal colonic mucosa samples. Briefly, DNA was quantified by real-time PCR and treated with bisulfite as for the MethyLight assay. Human sperm DNA and Universal methylated DNA (Chemicon, Temcula, CA) were included in each run as unmethylated and methylated controls, respectively. The bisulfite-converted DNA was probed at 1,505 individual CpG loci contained within 807 genes in the GoldenGate Methylation Cancer Panel I according to the manufacturer's instructions (Illumina). Hybridised arrays were scanned using the BeadArray Reader (Illumina). Extraction and normalization of intensity data was performed using the Beadscan software. To ensure adequate sample quality, only samples having > 75% loci with a detection *P*-value of < 0.05 were included for analysis.

### Statistical analysis

The methylation level at each CpG site, or β-value, was defined as the ratio of methylated allele to the sum of methylated and unmethylated alleles and ranged from 0 (completely unmethylated) to 1 (completely methylated). Normalisation of background intensity was estimated from a set of built-in negative controls and subtracted from each methylation data point as performed in other studies [23,24]. All statistical analyses were carried out using β-value as a continuous variable unless specified otherwise. To compare the number of methylated genes between different tumor subgroups, β-values were binarized using a methylated threshold of 0.297. Using this threshold, methylated controls in the array were classified as unmethylated at a 5% false discovery rate. A total of 84 CpG sites contained within 39 X-chromosome genes were excluded from the analysis in order to eliminate gender-specific bias.

Unsupervised and supervised hierarchical clustering analyses were performed with the *heatmap.2 *function in the *gplots *library. Unsupervised clustering was used to characterize methylation patterns in an unbiased fashion, as performed in other studies using methylation arrays [14,25-27]. Supervised clustering analysis was used to further investigate methylation differences observed in unsupervised clustering. The optimal number of clusters was determined using the Hubert & Levine internal cluster quality index [28]. The robustness of this number was evaluated by bootstrap resampling analysis (n = 1000). Additional evidence to support the delineation of clusters was obtained through unsupervised principal component analysis. The frequency and level of CpG methylation across different clusters was compared using a two-sample proportion test based on both binarised and continuous β-values. The association of clinicopathological and molecular variables with each cluster was analysed using continuous β-values and the two-sample proportion t-test. All statistical analyses were performed in R version 2.7.1 (The R Foundation for Statistical Computing) at 5% significance level unless otherwise stated. Where applicable, Bonferroni correction was applied to adjust for multiple testing.

## Results

### DNA methylation patterns in normal and tumor tissue

Unsupervised hierarchical clustering of DNA methylation data from 1,505 CpG sites in 28 samples of normal colonic mucosa revealed no distinct clusters [Additional file 1]. As expected, the methylation status of 84 CpG sites in 39 genes located in the X-chromosome was perfectly correlated with gender [Additional file 1]. These genes were excluded from subsequent analyses. For the 91 tumor samples, three clusters were observed when methylation data from all 1,505 loci were included in the analysis [Additional file 2].

**Figure 1:**
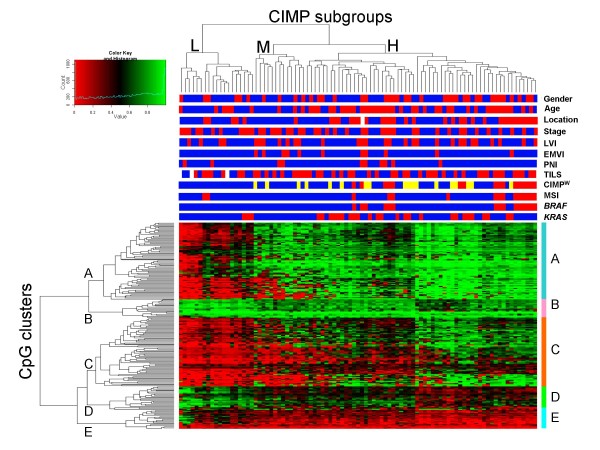
**Unsupervised hierarchical clustering of 202 tumor-specific probes (rows) in 91 CRC (columns)**. The 3 tumor clusters generated by this analysis were termed CIMP-High (CIMP-H), CIMP-Mid (CIMP-M) and CIMP-Low (CIMP-L). Clinicopathological and molecular features are shown above the heatmap. White rectangles are cases with missing data. Gender: female (red), male (blue); Age: ≥ 67 years (red), < 67 (blue); Tumor location: proximal (red), distal (blue); Tumor stage (ACPS): A or B (blue), C or D (red); Lymphovascular invasion (LVI): present (red), absent (blue); Extramural vascular invasion (EMVI): present (red), absent (blue); Perineural invasion (PNI): present (red), absent (blue); Tumor infiltrating lymphocytes (TILS): present (red), absent (blue); CIMP^W^: CIMP^W^-high (red), CIMP^W^-low (yellow), CIMP^W^-negative (blue); *BRAF*: mutant (red), wildtype (blue); *KRAS*: mutant (red), wildtype (blue); Microsatellite instability (MSI): positive (red), negative (blue). Five CpG clusters (A-E) were apparent from the analysis and showed differential methylation amongst the 3 CIMP subgroups.

A total of 202 CpG sites, corresponding to 132 genes (90 hypermethylated and 42 hypomethylated), were differentially methylated between tumor and normal colonic mucosa (*P *< 0.001, FDR 5%) [Additional file 3]. Unsupervised hierarchical clustering of methylation data from these 202 tumor-specific markers identified three major tumor groups (Fig. 1), referred to here as CIMP-high (CIMP-H; 59/91, 65%), CIMP-mid (CIMP-M; 13/91, 14%) and CIMP-low (CIMP-L; 19/91, 21%). The mean methylation level (β-value) of the 202 CpG sites for these groups was 0.617, 0.506 and 0.370, respectively (*P *< 0.001). Binarization of the methylation readings using a β-value cut-off of 0.297 revealed a decreasing number of methylated CpG sites for the three groups (167, 136 and 105 respectively; *P *< 0.001).

Although branching of the dendogram suggested the existence of two subgroups within CIMP-H (Fig. 1), the mean methylation level and the frequency of methylation between these groups were not significantly different (*P *= 0.37 and *P *= 0.90 respectively). Additional evidence for the validity of tumor segregation was obtained through unsupervised principal component analysis (PCA) [Additional file 4]. CIMP-H could be clearly segregated from CIMP-L. CIMP-H and CIMP-M could also be discriminated from each other, although less distinctly. This is presumably because of a greater similarity between these two groups [Additional file 4]. In further support, 3 was the most frequent optimal number of clusters in bootstrap resampling analysis.

### CIMP subgroups show distinctive clinicopathological and molecular features

The distribution of clinicopathological and molecular features for 91 CRC in relation to the methylation pattern obtained from analysis of all 202 differentially methylated CpG sites is shown in Fig. 1. Calculation of associations between these features and the three CIMP subgroups are shown in Table 1. Similar to previous reports on CIMP+, the CIMP-H tumors in this study were significantly associated with older age, proximal tumor location and *BRAF *mutation relative to CIMP-M and CIMP-L tumors. CIMP-H was also significantly associated with MSI+ when compared to CIMP-M, but not CIMP-L tumors. Two of the 15 MSI+ tumors were observed in the CIMP-L group and 13 in the CIMP-H group. Interestingly, the two patients with CIMP-L MSI+ tumors were aged 44 and 60 years, suggesting the underlying cause of the MSI+ phenotype was germline or somatic mutation of the mismatch repair genes rather than *hMLH1 *methylation. Indeed, no *hMLH1 *methylation was detected in both these cases in the GoldenGate Methylation Array data.

**Table 1:** Clinicopathological and molecular characteristics of CIMP subgroups.

	CIMP subgroup (n, %)			*P*		
		
	L	M	H	L *vs *M	M *vs *H	L *vs *H
	19 (21)	13 (14)	59 (65)			
Female	6 (32)	4 (31)	30 (51)			
Male	13 (68)	9 (69)	29 (49)	0.952	0.211	0.175
Age ≥ 67 years	6 (32)	5 (38)	37 (63)			
Age < 67 years	13 (68)	8 (62)	22 (37)	0.570	0.003	0.012
Proximal tumor site^1^	5 (26)	1 (8)	29 (49)			
Distal tumor site^2^	14 (74)	12 (92)	29 (49)	0.152	< 0.001	0.001
ACPS Stage A or B	8 (42)	4 (31)	36 (61)			
ACPS Stage C or D	11 (58)	9 (69)	23 (39)	0.520	0.025	0.105
LVI Negative	15 (79)	6 (46)	39 (66)			
LVI Positive	4 (21)	7 (54)	20 (34)	0.049	0.188	0.126
EMVI Negative	19 (100)	8 (62)	52 (88)			
EMVI Positive	0 (0)	5 (38)	7 (12)	0.005	0.031	0.024
PNI Negative	17 (89)	11 (85)	52 (88)			
PNI Positive	2 (11)	2 (15)	7 (12)	0.744	0.506	0.708
TILS Negative^3^	9 (47)	5 (38)	26 (44)			
TILS Positive	7 (37)	8 (62)	33 (56)	0.326	0.547	0.521
CIMP^W ^- negative^4^	19 (100)	10 (77)	28 (47)			
CIMP^W ^- low	0 (0)	3 (23)	15 (25)			
CIMP^W ^- high	0 (0)	0 (0)	16 (27)	1.000	< 0.001	< 0.001
MSI+	2 (11)	0 (0)	13 (18)			
MSI-	17 (89)	13 (100)	46 (78)	0.125	< 0.001	0.221
*BRAF *mutant	0 (0)	0 (0)	15 (25)			
*BRAF *wildtype	19 (100)	13 (100)	44 (75)	1.000	< 0.001	< 0.001
*KRAS *mutant	3 (16)	0 (0)	26 (44)			
*KRAS *wildtype	16 (84)	13 (100)	33 (56)	0.057	< 0.001	0.014

L, CIMP-low; M, CIMP-mid; H, CIMP-high; LVI, lymphovascular invasion; EMVI, extramural vascular invasion; PNI, perineural invasion; TILS, tumour infiltrating lymphocytes; CIMP^W^, classification of CIMP using the Weisenberger *et al *panel, whereby CIMP^W^-high is defined as 3 or more methylated loci, CIMP^W^-low as 1 or 2 methylated loci and CIMP^W^-negative as no methylated loci. MSI, microsatellite instability; ^1^Tumor location was unknown for 1 patient in CIMP-H, ^2^Number of distal CRC that were located at rectal site were 13, 1 and 11 in CIMP-L, CIMP-M and CIMP-H respectively, ^3^TILS data unknown for 3 patients in CIMP-L, ^4^*P *value for CIMP^W ^was generated from comparison between CIMP^W^-high and CIMP^W^-low or CIMP^W^-negative.

All 16 tumors classified as CIMP^W^-high by Methylight analysis using the panel of markers proposed by Weisenberger *et al *(>3/5 sites methylated) were contained within the CIMP-H group, while all 18 tumors classified as CIMP^W^-low (1/5 or 2/5 sites methylated) segregated into the CIMP-H or CIMP-M groups. Unfortunately, CpG sites for only 2 (*RUNX3*, *IGF2*) of the 5 genes in the CIMP^W ^panel were included in the Golden Gate arrays, thus preventing comparison of CIMP status by array and Methylight analysis. Nevertheless, there was good correlation between the array and Methylight methods for methylation levels of *RUNX3 *and *IGF2 *(all p < 0.05) using Pearson correlation.

All 15 tumors with *BRAF *mutation were CIMP-H. A significantly higher frequency of *KRAS *mutation was observed in CIMP-H compared to CIMP-L or CIMP-M tumors. None of the 13 CIMP-M tumors contained a *KRAS *mutation. The presence of extramural vascular invasion (EMVI) was more frequent in CIMP-M compared to CIMP-H or CIMP-L tumors. The presence of a tumor-infiltrating lymphocytic response (TILS) was not associated with any of the CIMP subgroups.

### Differentially methylated genes in CIMP subgroups

Five clusters of CpG loci, termed A to E, were apparent following unsupervised hierarchical clustering of methylation data for the 202 CpG loci that showed tumor-specific methylation (Fig. 1). CpG sites in clusters A and C were more highly methylated in CIMP-M and CIMP-H tumors compared to CIMP-L tumors, while the converse was true for the CpG sites in cluster D. CpG sites in cluster B and cluster E showed uniformly high and low methylation, respectively, in each of the 3 CIMP subgroups.

Using published data from studies on human stem cells [29], 50% (39/98) of the genes within clusters A and C were found to be targets for binding by Polycomb repressive complex 2 (PRC2) components and/or H3K27 trimethylation. In contrast, only 12% (5/41) of the genes within clusters B, D and E were targets (*P *< 0.001). These observations support previous reports that hypermethylated genes in cancer are frequent targets of PRC2-mediated H3K27 trimethylation [30].

## Discussion

The current study is the first to use array-based technology to enable comprehensive methylation profiling of CRC. A total of 1,505 CpG sites contained within 807 genes were assessed in 91 consecutive cases of CRC. The GoldenGate^® ^arrays employed here were recently used to profile methylation in head and neck cancer [31], renal cancer [32], glioblastoma [33] and hematological neoplasms [24,27]. The validity of these arrays for the quantitative assessment of methylation was shown in several previous studies by comparison with other quantitative methods [23,26,34]. The finding that methylation of CpG sites in X-linked genes correlated with gender provided further validation [Additional file 1]. Many of the genes found to be hypermethylated in this study were previously reported to be methylated in CRC [Additional file 5]. Finally, in agreement with earlier work on cancer [30], many of the genes showing *de novo *hypermethylation in this study of CRC (cluster A and C genes, Fig. 1) are known targets for PRC2 [29].

Similar to earlier studies in CRC that evaluated a limited number of methylation markers [3-10], comprehensive methylation profiling in the present study revealed the existence of distinct tumor subgroups (Fig. 1). The three major subgroups identified by unsupervised hierarchical clustering were classified as CIMP-H, CIMP-M and CIMP-L according to the level and frequency of methylation. In agreement with previous studies, CIMP-H tumors were associated with older patient age, proximal site and *BRAF *mutation (Table 1). All 16 tumors identified as CIMP^W^-high using a proposed consensus panel of 5 markers were contained within the CIMP-H group, as well as all 15 tumors containing a *BRAF *mutation. Using small numbers of methylation markers in unselected CRC, the original studies by Toyota *et al *reported CIMP+ frequencies of 62% [3] and 51% [35] whereas subsequent studies reported lower frequencies of 15-32% [5,7-10], [14]. In contrast, by investigating a large number of methylation sites and using unsupervised hierarchical clustering to analyze the results, we observed a relatively high proportion (65%) of CIMP-H tumors in the present study.

Previous studies have reported inconsistent results for the association between CpG island methylation and *KRAS *mutation [8,13,17,35,36], probably because of the different methylation markers used in each study. Analysis of a large number of CpG sites in the present study revealed that CIMP-H tumors showed a significantly higher *KRAS *mutation frequency compared to both CIMP-M and CIMP-L tumors (Table 1). This result agrees with some studies [4,5,8,35] but not others that found an inverse association between *KRAS *mutation and CIMP+ [7,9,10].

Since *BRAF *mutations are strongly associated with CIMP and mutually exclusive to *KRAS *mutations ([10]; Fig. 1), a point of interest is whether methylation patterns differ between tumors with *BRAF *and *KRAS *mutations. Supervised clustering analysis with Bonferroni correction revealed that only 1 of the 202 tumor-specific CpG sites was differentially methylated between these tumor groups (HTR1B_P222_F, upregulated in *BRAF *mutant tumors, *p *= 8.1 × 10^-6^). HTR1B (5-hydroxytryptamine (serotonin) receptor 1B) is a G protein-coupled multi-pass membrane protein involved in regulation of the serotonin system [37]. The gene is hypermethylated in lung cancer and its chromosome locus (16q14.1) is frequently deleted in a number of cancer types [38]. However, no links with BRAF or RAS mutations or signaling have been reported.

A novel finding of this array-based analysis was the existence of an apparent CIMP-M group (Fig. 1). These tumors displayed a higher frequency of EMVI compared to both CIMP-L and CIMP-H, and a significantly higher stage compared to CIMP-H (Table 1). CIMP-M tumors were almost exclusively located in the distal colon or rectum (12/13, 92%). MSI and *KRAS *and *BRAF *mutations were notably absent in these tumors, although this may be due to reportedly lower frequencies of these alterations in distal tumors [39]. Taken together, these results suggest CIMP-M tumors could be a distinct clinical and molecular entity, although confirmation in larger, independent tumor series is required.

After adjustment for multiple testing, 170 CpG sites were hypermethylated in CIMP-H compared to CIMP-L. The 112 genes containing these CpG sites are ranked according to significance in Additional file 5. Of these, 54 were previously reported as methylated in cancer, 38 as methylated in gastrointestinal cancers and 30 in CRC [Additional file 5]. Of the top 10 genes that were hypermethylated in CIMP-H compared to CIMP-L tumors, 5 have previously been implicated in the pathogenesis of gastrointestinal tumors (*NTRK3*, *HS3ST2*, *TWIST1*, *CD40 *and *EYA4*). Somatic mutation of *NTRK3 *has been reported in human colon cancer [40], while methylation of *EYA4 *has been documented previously in ulcerative colitis-associated dysplasia [41] and CRC [42].

CIMP-M tumors were found to have a relatively high incidence of EMVI (38%) compared to CIMP-H and CIMP-L tumors (Table 1). Supervised analysis revealed that *HS3ST2*, also known as *3-OST-2*, was the only gene to be differentially methylated between tumors showing presence or absence of EMVI. Methylation-associated silencing of *HS3ST2 *expression has been demonstrated in breast, lung, pancreatic and colon cancers [43]. This gene encodes an enzyme that modifies heparin sulfate proteoglycans [44] involved in cell adhesion and migration [45], thus suggesting a possible mechanistic link between *HS3ST2 *methylation and EMVI.

The use of Illumina GoldenGate^® ^Beadarray technology in this study allowed a large number of CpG sites to be evaluated for methylation in an unbiased fashion. However, there are several limitations with this approach for the characterization of CIMP subgroups in CRC. Firstly, only a small fraction of all genes were investigated for methylation and in 70% of these just one CpG site per gene was evaluated. Secondly, it is unclear whether the methylation level at these sites relates to expression of the genes. Thirdly, some of the probes used in this assay contain single nucleotide polymorphisms (SNPs) or repetitive elements that could influence methylation analysis [25]. The cost effectiveness of using arrays to characterize CIMP-H is questionable, given the strong concordance between CIMP-H from this study and CIMP^w^. Further studies should clarify if the additional information provided by methylation arrays is worth the complexity and expense.

## Conclusions

Methylation profiling of 807 cancer-related genes revealed the presence of three CRC subgroups with distinct clinicopathological and molecular features. Similar to earlier studies that investigated fewer methylation markers, CIMP-H CRC were associated with older patient age, proximal location and mutations in *BRAF *and *KRAS*. Further investigations in large and independent population-based series are required to validate these findings and to assess the clinical utility of CIMP subgroups.

## Competing interests

The authors declare that they have no competing interests.

## Authors' contributions

PWA, FG, NL, PLL, AV carried out the experimental work. ML performed the statistical analysis. CP and WPY contributed gave critical clinical perspective to the results, and BI and RS co-ordinated the study and compiled the manuscript. All authors read and approved the final version of the manuscript.

## Pre-publication history

The pre-publication history for this paper can be accessed here:

http://www.biomedcentral.com/1471-2407/10/227/prepub

## Supplementary Material

Additional file 1 **Unsupervised hierarchical clustering of 1505 probes (rows) in 28 normal colonic tissues (columns)**. Methylation of X-chromosome genes (enclosed within yellow rectangle) showed 100% correlation to gender as indicated by the red (female) and blue (male) bar above the heatmap. Click here for file

Additional file 2 **Unsupervised hierarchical clustering of 1505 probes (rows) in 91 colorectal tumors (columns)**. Three tumor subgroups were revealed when methylation data from all 1,505 loci were analysed. Click here for file

Additional file 3 **202 CpG sites differentially methylated between normal and tumour tissues**. Methylation status at 202 CpG loci differentially methylated between normal and tumour tissues and presence of repetitive element or single nucleotide polymorphisms within probes. Click here for file

Additional file 4 **Principal component analysis of 202 CpG loci that were differentially methylated between tumor and normal colonic tissue**. This identified principal component 2 as the top ranking dimension and which explained 20% of the variability in the dataset. CIMP-H tumors are denoted in green, CIMP-M in black and CIMP-L in red. Click here for file

Additional file 5 **112 genes differentially methylated between CIMP-H and CIMP-L**. Known functions of 112 genes differentially methylated between CIMP-H and CIMP-L and their reported methylation in cancer and putative roles in gastrointestinal cancer. Click here for file
