# Supplementary figures and images for: Comprehensive profiling of DNA methylation in colorectal cancer reveals subgroups with distinct clinicopathological and molecular features
Source: BMC Cancer. 2010 May 21;10:227. doi: 10.1186/1471-2407-10-227 (PMC2880997; doi:10.1186/1471-2407-10-227)

## Slide 1
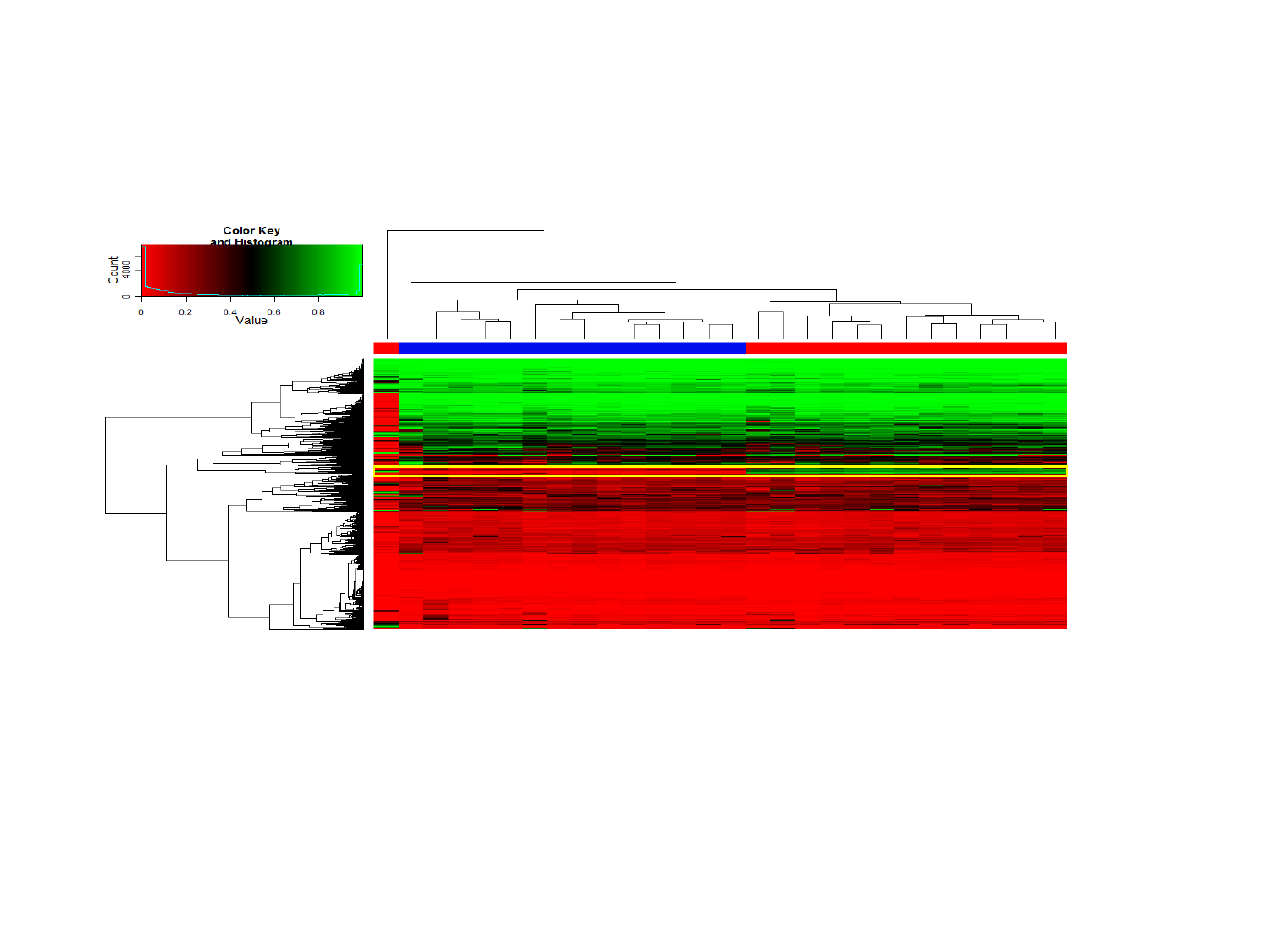

Supplement: Additional file 1 — Unsupervised hierarchical clustering of 1505 probes (rows) in 28 normal colonic tissues (columns). Methylation of X-chromosome genes (enclosed within yellow rectangle) showed 100% correlation to gender as indicated by the red (female) and blue (male) bar above the heatmap. [file 1471-2407-10-227-S1.PPT]

## Slide 1
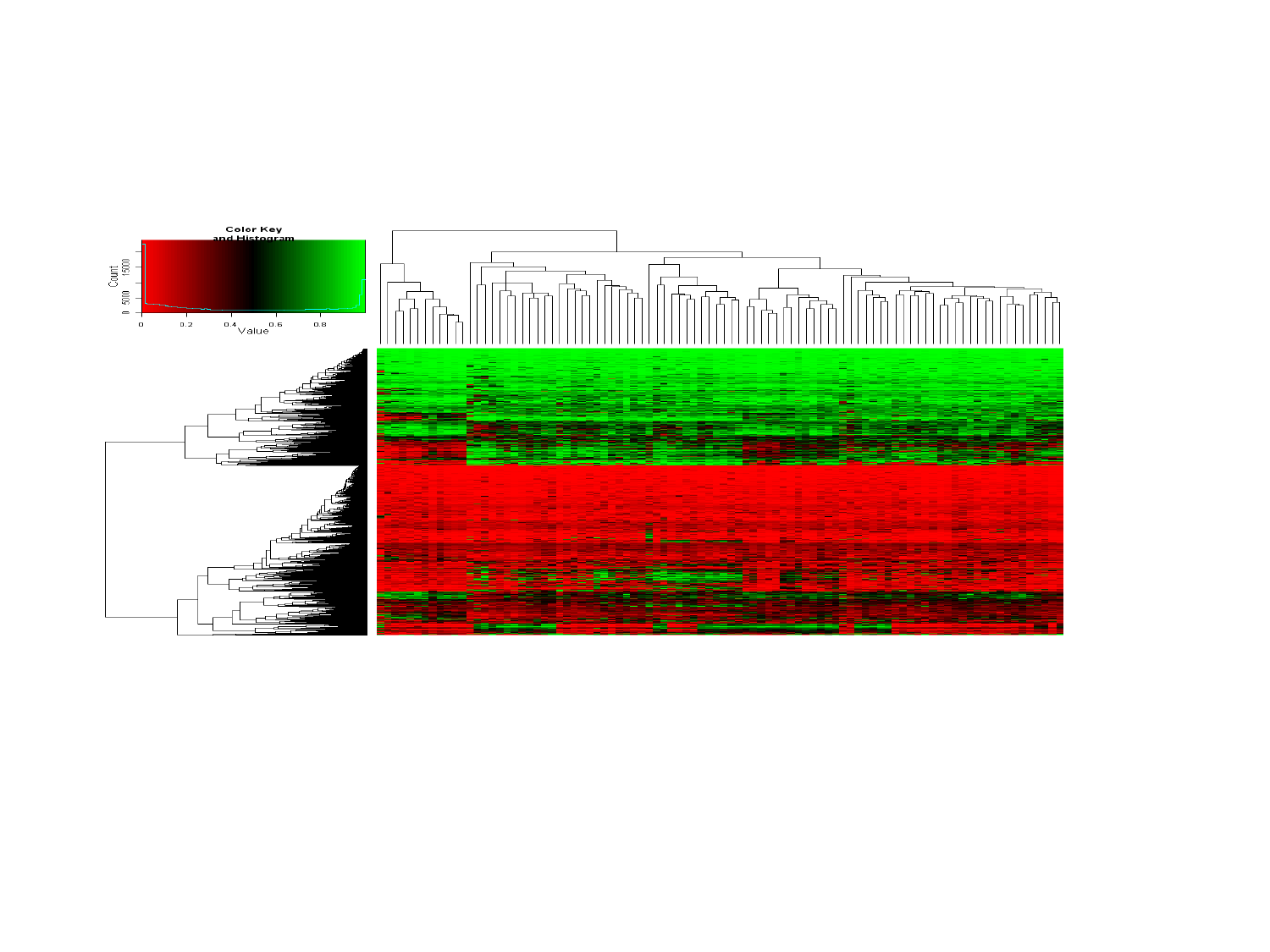

Supplement: Additional file 2 — Unsupervised hierarchical clustering of 1505 probes (rows) in 91 colorectal tumors (columns). Three tumor subgroups were revealed when methylation data from all 1,505 loci were analysed. [file 1471-2407-10-227-S2.PPT]

## Slide 1
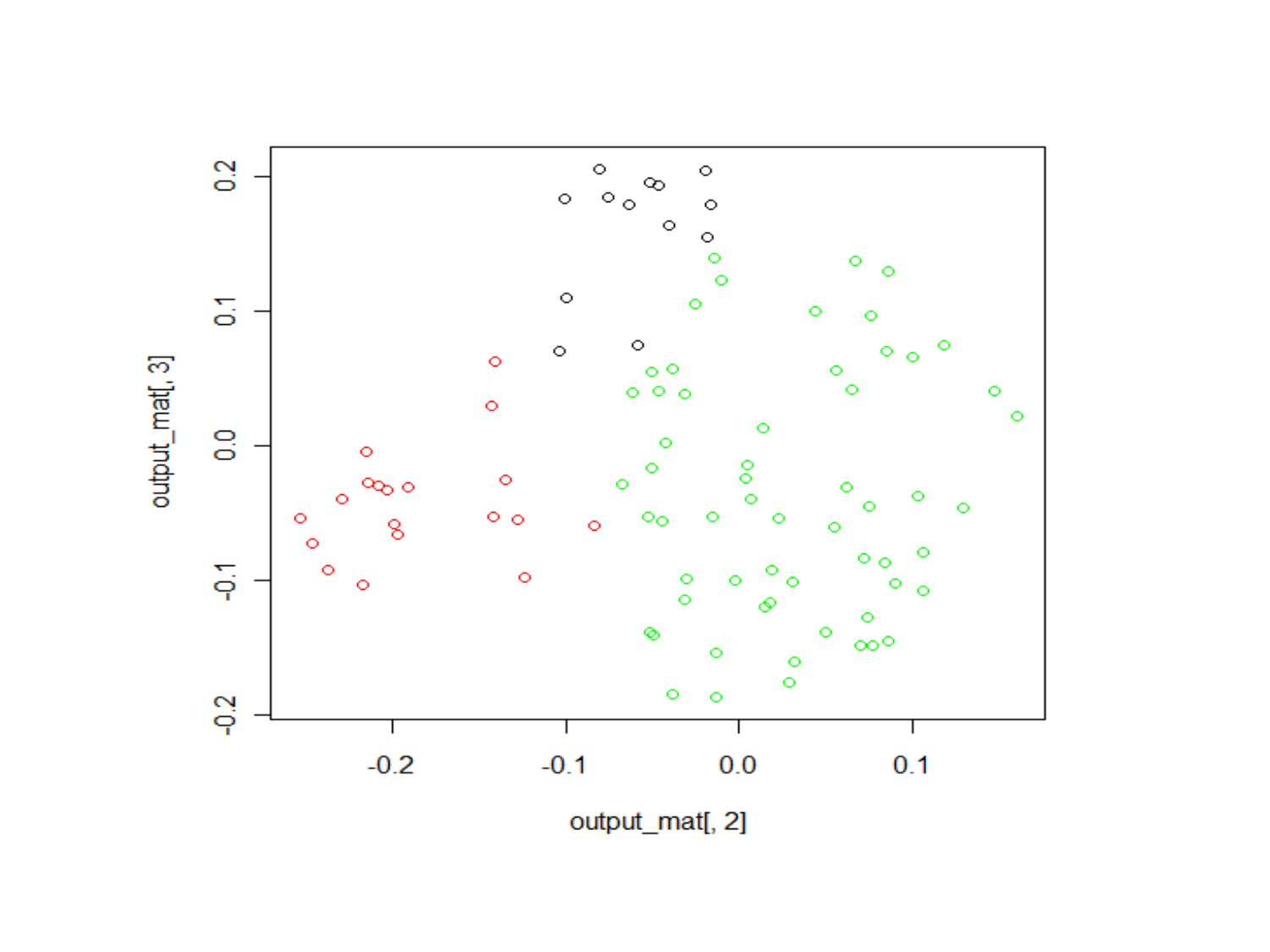

Supplement: Additional file 4 — Principal component analysis of 202 CpG loci that were differentially methylated between tumor and normal colonic tissue. This identified principal component 2 as the top ranking dimension and which explained 20% of the variability in the dataset. CIMP-H tumors are denoted in green, CIMP-M in black and CIMP-L in red. [file 1471-2407-10-227-S4.PPT]
